# Beauty Standards and Societal Influences on Facelift Surgery Decisions in Saudi Arabia

**DOI:** 10.7759/cureus.80041

**Published:** 2025-03-04

**Authors:** Sari M Rabah, Hussam F Alkhars, Abdullah Q AlAlwan, Talal A Albalawi, Hana A Alazzmi, Maha AlQahtani, Ali F Alkhars

**Affiliations:** 1 Plastic and Reconstructive Surgery, King Abdulla Bin Abdulaziz University Hospital, Princess Nourah Bint Abdulrahman University, Riyadh, SAU; 2 Medicine, King Faisal University, Alhofuf, SAU; 3 Plastic and Reconstructive Surgery, King Faisal University, Al Ahsa, SAU; 4 Plastic and Reconstructive Surgery, Saudi Board of Plastic Surgery, King Abdulla Bin Abdulaziz University Hospital, Princess Nourah Bint Abdulrahman University, Riyadh, SAU; 5 Surgery, College of Medicine, King Saud University, Riyadh, SAU; 6 Medicine, King Faisal University, Al Ahsa, SAU

**Keywords:** attitude, facelift surgery, motives, perception, population, saudi arabia

## Abstract

Introduction

Facelifting, or rhytidectomy, is a surgical procedure to reduce facial wrinkles and create a youthful appearance. This study aims to explore the factors influencing the decision to undergo facelift surgery in Saudi Arabia, focusing on beauty standards and cultural expectations.

Methods

We conducted a cross-sectional study from January to December 2024, recruiting Saudi individuals 18 years or older. We developed and validated a questionnaire in both English and Arabic, which was distributed via social media platforms and healthcare networks. Data were analyzed using IBM SPSS Statistics for Windows, Version 27.0 (IBM Corp., Armonk, NY), with categorical variables summarized as frequencies and percentages. Associations between demographic variables and attitudes toward facelift surgery were assessed using the Chi-square test, with statistical significance set at p < 0.05.

Results

Among 726 participants, most were 36 to 45 years (n=195, 26.9%) and male (n=379, 52.2%). Most held a postgraduate degree (n=284, 39.1%) and were employed (n=352, 48.5%). Overall, 39% (n=283) agreed or strongly agreed with undergoing facelift surgery, citing self-confidence (n=283, 39%) and societal beauty standards (n=287, 39.5%) as key motivations. Social media influence (n=294, 40.5%) and plastic surgeon consultations (n=296, 40.8%) significantly shaped perceptions. Participants of >55 years were more likely to undergo facelifts to feel young again (n=87, 50.6%) compared to those aged 18 to 25 years (n=32, 30.2%, p = 0.048).

Conclusions

This study highlights multiple factors influencing attitudes toward facelift surgery, including societal beauty standards, media influence, and professional consultations. While findings align with global trends, the perception of facelifts as procedures primarily for older adults suggests unique cultural influences. These insights are crucial for healthcare providers and policymakers to address the growing demand for cosmetic procedures while ensuring informed decision-making. Further research should explore long-term trends in cosmetic surgery within Saudi Arabia.

## Introduction

Facelifting, or rhytidectomy, is a surgical procedure to reduce facial wrinkles to create a more youthful appearance. Although its history dates back over a century, the procedure has gained increasing popularity in recent decades due to societal emphasis on youthfulness in middle and older age groups [1]. In 1976, Mitz and Peyronie defined the superficial musculoaponeurotic system, a key anatomical structure in facelift surgery [2]. In the late 1980s and early 1990s, Hamra refined Skoog’s technique by introducing deep plane rhytidectomy and the composite facelift to enhance the periorbital and nasolabial regions [3]. Owsley further improved this approach by incorporating malar fat pad dissection and suspension to enhance the nasolabial crease [4]. Ramirez later introduced the subperiosteal rhytidectomy technique to address the cheek, forehead, jowls, lateral canthus, and eyebrows [5].

Patients primarily seek facelift procedures to achieve a younger, more refreshed, and attractive appearance. To enhance outcomes, surgeons have developed various operative modifications and adjunctive procedures [6-9]. Patient-reported outcome studies using validated surveys have demonstrated high satisfaction rates [10]. Similarly, studies evaluating surgeon assessments and public perceptions indicate that facelifting effectively reduces the appearance of aging [11]. Previous research has identified factors influencing the decision to undergo cosmetic procedures, including increased media exposure and low self-esteem [12]. A study in Saudi Arabia reported that 65.7% of individuals visiting cosmetic clinics were motivated by exposure to before-and-after surgery photographs [13]. Given these findings, we conducted this study to explore the factors influencing the decision to undergo facelift surgery in Saudi Arabia, focusing on beauty standards and cultural expectations.

## Materials and methods

We conducted this cross-sectional study from January to December 2024 and recruited Saudi individuals from various regions. We included men and women of 18 years or older who resided in Saudi Arabia. We excluded individuals younger than 18 years or those residing outside Saudi Arabia. Throughout the study, we maintained participant privacy and data confidentiality. The Research Ethics Committee of King Faisal University approved the study (reference code: KFU-REC-2025-FEB-ETHICS3070).

We initially developed the study questionnaire in English to ensure clarity in medical terminology. Afterward, we collaborated with a language expert to translate the questionnaire into Arabic, facilitating comprehension among Arabic-speaking respondents. We consulted subject matter experts who reviewed and provided feedback to validate the questionnaire. We assessed content validity using Lawshe’s method and removed items with a content validity ratio below 0.99. We then conducted a pilot study with 102 participants to assess reliability, though we excluded pilot study data from the final analysis.

We used non-probability convenience sampling to recruit participants who met the inclusion criteria. We distributed the Arabic version of the questionnaire via a Google Form Survey on social media platforms and healthcare networks. The questionnaire consisted of three sections: (1) demographic characteristics, (2) general perceptions of facelift surgery and factors influencing the decision to undergo the procedure, and (3) previous facelift experiences and associated influencing factors. We compiled responses in Microsoft Excel (Microsoft Corp., Redmond, WA) for preliminary assessment.

Data analysis

We performed statistical analysis using IBM SPSS Statistics for Windows, Version 27.0 (IBM Corp., Armonk, NY). We summarized participants’ demographic characteristics using descriptive statistics. We reported categorical variables as frequencies and percentages. To assess perceptions and attitudes toward facelift surgery, we used a five-point Likert scale (strongly disagree, disagree, neutral, agree, strongly agree) and analyzed the distribution of responses. We examined the association between gender and attitudes toward facelift surgery using the Chi-square (Pearson X²) test and applied exact probability tests where appropriate. Similarly, we analyzed associations between age groups and attitudes toward facelift surgery using the Chi-square test and exact probability tests to determine significant differences. We set statistical significance at p < 0.05.

## Results

Table 1 presents the demographic characteristics of the 726 participants. Most participants resided in the Central region (n=209, 28.8%), followed by the Western (n=162, 22.3%), Eastern (n=159, 21.9%), Southern (n=103, 14.2%), and Northern regions (n=93, 12.8%). The majority were 36 to 45 years (n=195, 26.9%), followed by 46 to 55 years (n=173, 23.8%) and 26 to 35 years (n=167, 23.0%). Men comprised 52.2% of the sample (n=379). Most participants held a postgraduate degree (n=284, 39.1%), while 48.5% were employed (n=352). The most common income range was 10,000-20,000 Saudi Riyals (n=251, 34.6%), and 50.3% were married (n=365).

**Table 1:** Biodemographic characteristics of study participants in Saudi Arabia (N=726) The data are represented as (N) and (%) for participants in each question. Abbreviation: SR, Saudi Riyals.

Biodemographic data		N	Percent
Residence region	Central region	209	28.8
	Northern region	93	12.8
	Eastern region	159	21.9
	Western region	162	22.3
	Southern region	103	14.2
Age in years	18-25	106	14.6
	26-35	167	23.0
	36-45	195	26.9
	46-55	173	23.8
	> 55	85	11.7
Gender	Male	379	52.2
	Female	347	47.8
Educational level	No formal education	75	10.3
	General education	73	10.1
	Diploma	156	21.5
	University education	138	19.0
	Post-graduate	284	39.1
Employment status	Not working	198	27.3
	Student	176	24.2
	Employed	352	48.5
Monthly income	< 5,000 SR	130	17.9
	5,000-10,000 SR	231	31.8
	10,000-20,000 SR	251	34.6
	> 20,000 SR	114	15.7
Marital status	Single	253	34.8
	Married	365	50.3
	Divorced/widow	108	14.9

Table 2 presents participants’ perceptions and attitudes toward facelift surgery. Overall, 39% (n=283) agreed or strongly agreed with undergoing the procedure, with key motivations including self-confidence (n=283, 39%) and societal aesthetic standards (n=287, 39.5%). Plastic surgeon consultations (n=295, 40.7%) and social media influence (n=294, 40.5%) also significantly shaped opinions. Participants cited correcting facial deformities (n=246, 33.9%) and bullying experiences (n=243, 33.6%) as additional motivating factors.

**Table 2:** Perceptions and attitudes of study participants toward facelift surgery in Saudi Arabia (N=726) The data are represented as (N) and (%) for participants in each question.

Questions/Statements	Participant responses									
	Strongly disagree		Disagree		Neutral		Agree		Strongly agree	
	N	Percent	N	Percent	N	Percent	N	Percent	N	Percent
What do you think about facelift surgery?	85	11.7	194	26.7	164	22.6	194	26.7	89	12.3
A person performs facelift surgery to correct facial deformities.	116	16.0	183	25.2	181	24.9	165	22.7	81	11.2
A person performs a facelift because of bullying from his family and those close to him.	110	15.2	172	23.7	200	27.5	158	21.8	86	11.8
A person gets a facelift because social media celebrities' influence.	84	11.6	178	24.5	170	23.4	175	24.1	119	16.4
People's desire to undergo facelift increases when they consult plastic surgeons and obtain approval from them.	85	11.7	191	26.3	155	21.3	198	27.3	97	13.4
A person performs a facelift because of an aesthetic standard in society.	84	11.6	194	26.7	161	22.2	200	27.5	87	12.0
A person performs a facelift to increase self-confidence.	87	12.0	177	24.4	179	24.7	190	26.2	93	12.8
Facelift surgeries are usually performed in elderlies.	104	14.3	190	26.2	182	25.1	171	23.6	79	10.9
A person performs facelift procedures to feel young again.	89	12.3	166	22.9	184	25.3	204	28.1	83	11.4

**Figure 1:**
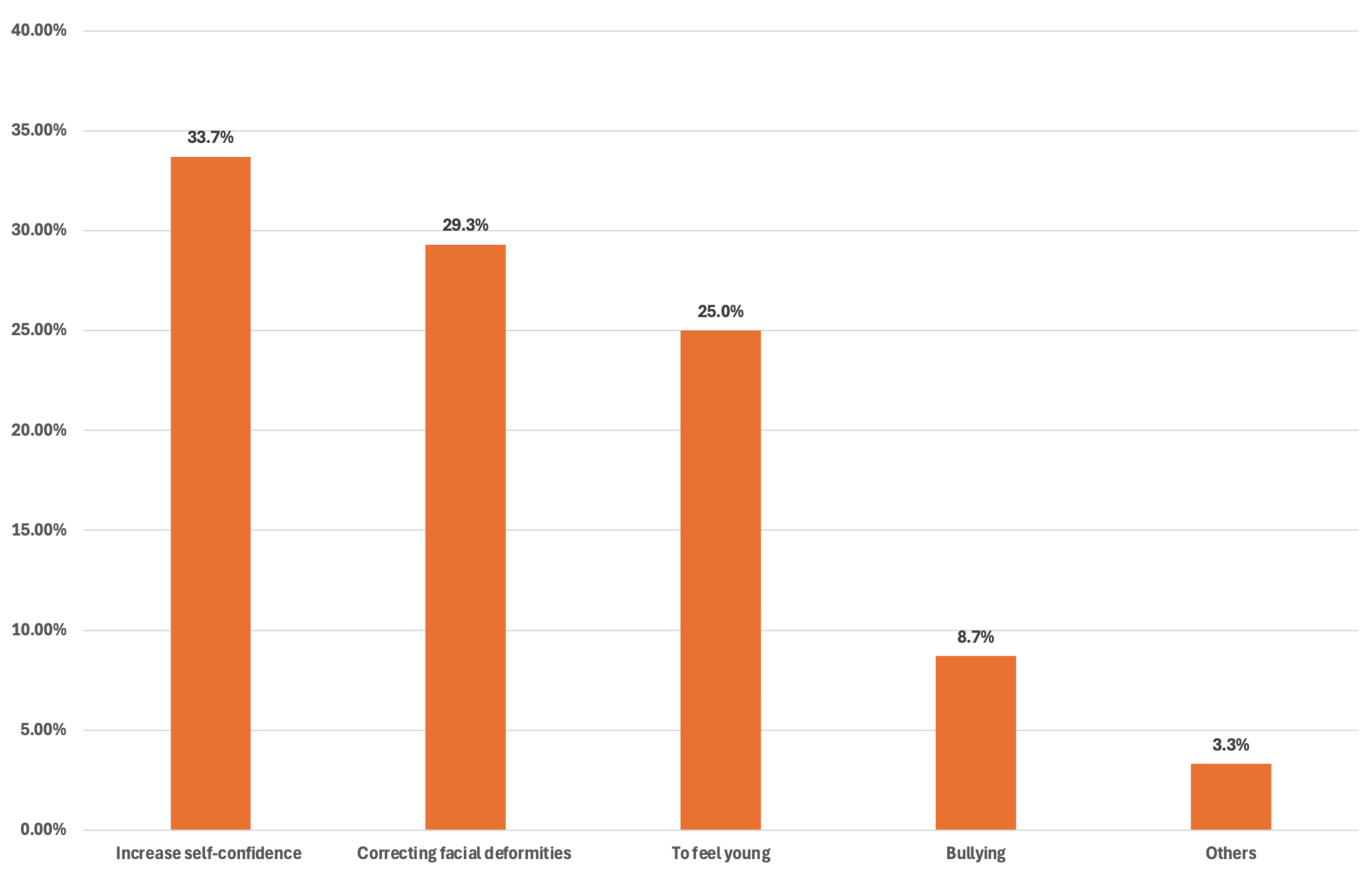
Frequency of and motives behind undergoing facelift surgery among the study participants The data are represented as (%).

Figure 1 presents the frequency and motives related to facelift. A portion of 92 (12.7%) had undergone facelift surgery. Among those who had undergone the procedure, the most common reasons for seeking facelift included cosmetic or emotional factors, such as increasing their self-confidence (33.7%). Medical issues, such as correcting facial deformities (29.3%). individuals also cited to feel young (25.0%). Additionally, bullying was a factor for another (8.7%) of respondents and (3.3%) responded with other reasons.

Table 3 compares attitudes toward facelift surgery by gender. While men (n=300, 41.4%) were slightly more likely than women (n=263, 36.3%) to support the procedure, no statistically significant gender differences emerged (p > 0.05). Social media influence, plastic surgeon recommendations, and aesthetic standards affected both genders similarly.

**Table 3:** Distribution of participants' attitudes toward facelift surgery by gender ^a^Pearson X^2^ test ^b^Exact probability test

Attitude question/statement	Response choice	Gender				P-value
		Male		Female		
		N	Percent	N	Percent	
What do you think about facelift surgery?	Disagree	138	36.4	141	40.6	0.347^a^
	Neutral	84	22.2	80	23.1	
	Agree	157	41.4	126	36.3	
A person performs facelift surgery to correct facial deformities.	Disagree	157	41.4	142	40.9	0.968^a^
	Neutral	93	24.5	88	25.4	
	Agree	129	34.0	117	33.7	
A person performs a facelift because of bullying from his family and those close to him.	Disagree	149	39.3	133	38.3	0.266^a^
	Neutral	112	29.6	88	25.4	
	Agree	118	31.1	126	36.3	
A person gets a facelift because of social media celebrities’ influence.	Disagree	137	36.1	125	36.0	0.967^a^
	Neutral	90	23.7	80	23.1	
	Agree	152	40.1	142	40.9	
People's desire to undergo facelift increases when they consult plastic surgeons and obtain approval from them.	Disagree	140	36.9	136	39.2	0.186^b^
	Neutral	91	24.0	64	18.4	
	Agree	148	39.1	147	42.4	
A person performs a facelift because of an aesthetic standard in society.	Disagree	152	40.1	126	36.3	0.574^b^
	Neutral	82	21.6	79	22.8	
	Agree	145	38.3	142	40.9	
A person performs a facelift to increase self-confidence.	Disagree	127	33.5	137	39.5	0.236^a^
	Neutral	96	25.3	83	23.9	
	Agree	156	41.2	127	36.6	
Facelift surgeries are usually performed in elderlies.	Disagree	144	38.0	150	43.2	0.264^a^
	Neutral	103	27.2	79	22.8	
	Agree	132	34.8	118	34.0	
A person performs facelift procedures to feel young again.	Disagree	135	35.6	120	34.6	0.648^a^
	Neutral	100	26.4	84	24.2	
	Agree	144	38.0	143	41.2	

Table 4 examines attitudes by age group. Participants >55 years were significantly more likely to consider facelift surgery to feel young again (n=87, 50.6%) compared to the 18-25 age group (n=32, 30.2%, p = 0.048). Additionally, attitudes toward facelifts for correcting facial deformities varied significantly across age groups (p = 0.001), with the 36-45 age group showing the lowest disagreement (n=67, 34.4%). No other significant age-related differences were observed.

**Table 4:** Distribution of participants' attitudes toward facelift surgery according to age group ^a^Exact probability test *P < 0.05 (significant)

Attitude question/statement	Response choice	Age in years										P-Value^a^
		18-25		26-35		36-45		46-55		> 55		
		N	Percent	N	Percent	N	Percent	N	Percent	N	Percent	
What do you think about facelift surgery?	Disagree	43	40.6	66	39.5	67	34.4	66	38.2	37	43.5	0.827
	Neutral	24	22.6	39	23.4	45	23.1	42	24.3	14	16.5	
	Agree	39	36.8	62	37.1	83	42.6	65	37.6	34	40.0	
A person performs facelift surgery to correct facial deformities.	Disagree	55	51.9	78	46.7	67	34.4	69	39.9	30	35.3	0.001*
	Neutral	27	25.5	30	18.0	67	34.4	35	20.2	22	25.9	
	Agree	24	22.6	59	35.3	61	31.3	69	39.9	33	38.8	
A person performs a facelift because of bullying from his family and those close to him.	Disagree	38	35.8	62	37.1	82	42.1	67	38.7	33	38.8	0.371
	Neutral	27	25.5	43	25.7	47	24.1	52	30.1	31	36.5	
	Agree	41	38.7	62	37.1	66	33.8	54	31.2	21	24.7	
A person gets a facelift because of social media celebrities’ influence.	Disagree	39	36.8	60	35.9	73	37.4	60	34.7	30	35.3	0.965
	Neutral	20	18.9	42	25.1	48	24.6	40	23.1	20	23.5	
	Agree	47	44.3	65	38.9	74	37.9	73	42.2	35	41.2	
People's desire to undergo facelift increases when they consult plastic surgeons and obtain approval from them.	Disagree	48	45.3	69	41.3	63	32.3	65	37.6	31	36.5	0.271
	Neutral	21	19.8	32	19.2	44	22.6	44	25.4	14	16.5	
	Agree	37	34.9	66	39.5	88	45.1	64	37.0	40	47.1	
A person performs a facelift because of an aesthetic standard in society.	Disagree	42	39.6	67	40.1	73	37.4	57	32.9	39	45.9	0.513
	Neutral	27	25.5	31	18.6	42	21.5	45	26.0	16	18.8	
	Agree	37	34.9	69	41.3	80	41.0	71	41.0	30	35.3	
A person performs a facelift to increase self-confidence.	Disagree	41	38.7	61	36.5	74	37.9	53	30.6	35	41.2	0.503
	Neutral	23	21.7	47	28.1	48	24.6	40	23.1	21	24.7	
	Agree	42	39.6	59	35.3	73	37.4	80	46.2	29	34.1	
Facelift surgeries are usually performed in elderlies.	Disagree	48	45.3	62	37.1	78	40.0	74	42.8	32	37.6	0.926
	Neutral	24	22.6	44	26.3	48	24.6	45	26.0	21	24.7	
	Agree	34	32.1	61	36.5	69	35.4	54	31.2	32	37.6	
A person performs facelift procedures to feel young again.	Disagree	46	43.4	62	37.1	73	37.4	54	31.2	20	23.5	0.048*
	Neutral	28	26.4	42	25.1	44	22.6	48	27.7	22	25.9	
	Agree	32	30.2	63	37.7	78	40.0	71	41.0	43	50.6	

## Discussion

This study examined perceptions and attitudes toward facelift surgery among participants from various regions of Saudi Arabia. The demographic distribution showed that most participants were middle-aged men with varying levels of education, employment, and income. These diverse demographics provide valuable insights into attitudes toward facelift surgery.

The findings indicate that participants generally viewed facelifts positively, particularly for correcting facial deformities and enhancing self-confidence. This aligns with global trends, where cosmetic procedures are recognized for aesthetic improvement and psychological and functional benefits. Studies in the United States highlight that individuals often pursue facelifts to improve their quality of life, especially those with visible facial differences or low self-esteem [14]. Similarly, in South Korea, where cosmetic procedures are widely accepted, facelifts are commonly associated with personal and professional growth [15].

Social media influence emerged as a significant factor shaping perceptions of facelift surgery. A substantial proportion of participants acknowledged the role of social media influencers in promoting cosmetic procedures. This reflects global trends, as platforms like Instagram and TikTok have normalized cosmetic enhancements, particularly among younger audiences. This finding is consistent with previous research indicating that exposure to idealized images on social media can lead to a higher interest in cosmetic surgery [16].

Professional consultations with plastic surgeons also played a crucial role in shaping attitudes. Many participants reported that consultations influenced their perceptions of facelifts, highlighting the importance of professional guidance. A study in Australia emphasizes the role of informed consultations in improving patient satisfaction and setting realistic expectations [17]. These findings suggest that plastic surgeons in Saudi Arabia should prioritize patient education to ensure informed decision-making.

Many participants associated facelifts primarily with older individuals, differing from trends in other countries. In South Korea and the United States, individuals in their 30s and 40s increasingly undergo facelifts as a preventative measure against aging [18,19]. This cultural difference may reflect unique societal attitudes toward aging in Saudi Arabia.

Study limitations

This study stands out as it measures the factors influencing the decision to undergo facelift surgery in Saudi Arabia. However, this study has several limitations. The sample distribution across different regions of Saudi Arabia was uneven. Additionally, reliance on an online survey limited participation to individuals with internet access, potentially affecting the generalizability of the findings. Self-reporting bias may have influenced responses, as participants might have provided socially desirable answers rather than their true opinions. The study also lacks clinical validation, as it focuses on self-reported attitudes rather than actual facelift procedures performed. Furthermore, unmeasured factors, such as satisfactory results, pain and recovery from the procedure, the social recovery, religious beliefs, psychological motivations, and economic considerations, may have influenced participants’ perceptions. The cross-sectional design captures attitudes at a single point in time, making it difficult to assess changes over time or establish causal relationships. Future research should address these limitations by incorporating longitudinal designs, clinical data, and a more diverse sample.

## Conclusions

This study highlights multiple factors influencing attitudes toward facelift surgery, including societal beauty standards, media influence, professional consultations, and personal motivations. While the findings align with global trends, the perception of facelifts as procedures primarily for older adults suggests unique cultural influences. These insights are crucial for healthcare providers and policymakers to address the growing demand for cosmetic procedures while ensuring informed decision-making. Future research should explore these attitudes further and assess the long-term impact of cosmetic surgery trends in Saudi Arabia.
